# Advances in molecular dynamics approaches for investigating cell-penetrating peptides

**DOI:** 10.1007/s12551-026-01409-y

**Published:** 2026-02-10

**Authors:** Eric Catalina-Hernandez, Alex Peralvarez-Marin

**Affiliations:** 1 Unit of Biophysics, Department of Biochemistry and Molecular Biology, Facultat de Medicina, Universitat Autònoma de Barcelona, Av. Can Domènech S/N, 08193 Cerdanyola del Vallès, Catalonia Spain; 2 Institute of Neurosciences, Universitat Autònoma de Barcelona, 08193 Cerdanyola del Vallès, Catalonia Spain

**Keywords:** Membrane active peptide, Cell-penetrating peptide, Molecular dynamics, Peptide-membrane interaction, Enhanced sampling

## Abstract

Membrane active peptides (MAPs) are short, cationic peptides capable of interacting with biological membranes, often altering their structure or function. Two of the most important classes of MAPs are cell-penetrating peptides (CPPs), which can translocate cellular membranes without causing cytotoxicity, and antimicrobial peptides (AMPs), peptides that can disrupt microbial membranes through pore formation or membrane lysis. Despite extensive experimental research, a comprehensive understanding of CPPs’ mechanism at the molecular level remains elusive. Molecular dynamics (MD) simulations offer a powerful approach to investigate protein-lipid interactions and the dynamic behavior of peptide-membrane systems at atomic resolution. Nonetheless, capturing these complex processes through conventional MD simulations is computationally demanding. To address this, a range of enhancing sampling techniques has been developed. In this review, we discuss the MD methodologies available for studying CPPs’ interactions with membranes, focusing on the techniques introduced by our group. Our aim is to provide a useful reference for future investigations into peptide-membrane interactions, ultimately advancing molecular-level insight into these biologically significant systems.

## Introduction

Biological membranes are fundamental structures within cells and organelles, crucial for compartmentalization and regulating molecular transport and signaling (Cooper 2000). The cell membrane separates the internal cellular environment from the extracellular space, maintaining the integrity of intracellular components while preventing the entry of foreign substances. These membranes also function as selective barriers, controlling the passage of molecules into and out of the cell. Small organic compounds with appropriate hydrophobic and hydrophilic properties, such as small hydrophobic molecules, can traverse the membrane passively. Larger or charged molecules typically require specialized transport mechanisms (Pei 2022). Beyond their structural role, membranes serve as dynamic platforms for a wide array of biochemical processes, coordinating material exchange, cellular signaling, and energy transduction (Cheng And Smith 2019).

**Fig. 1:**
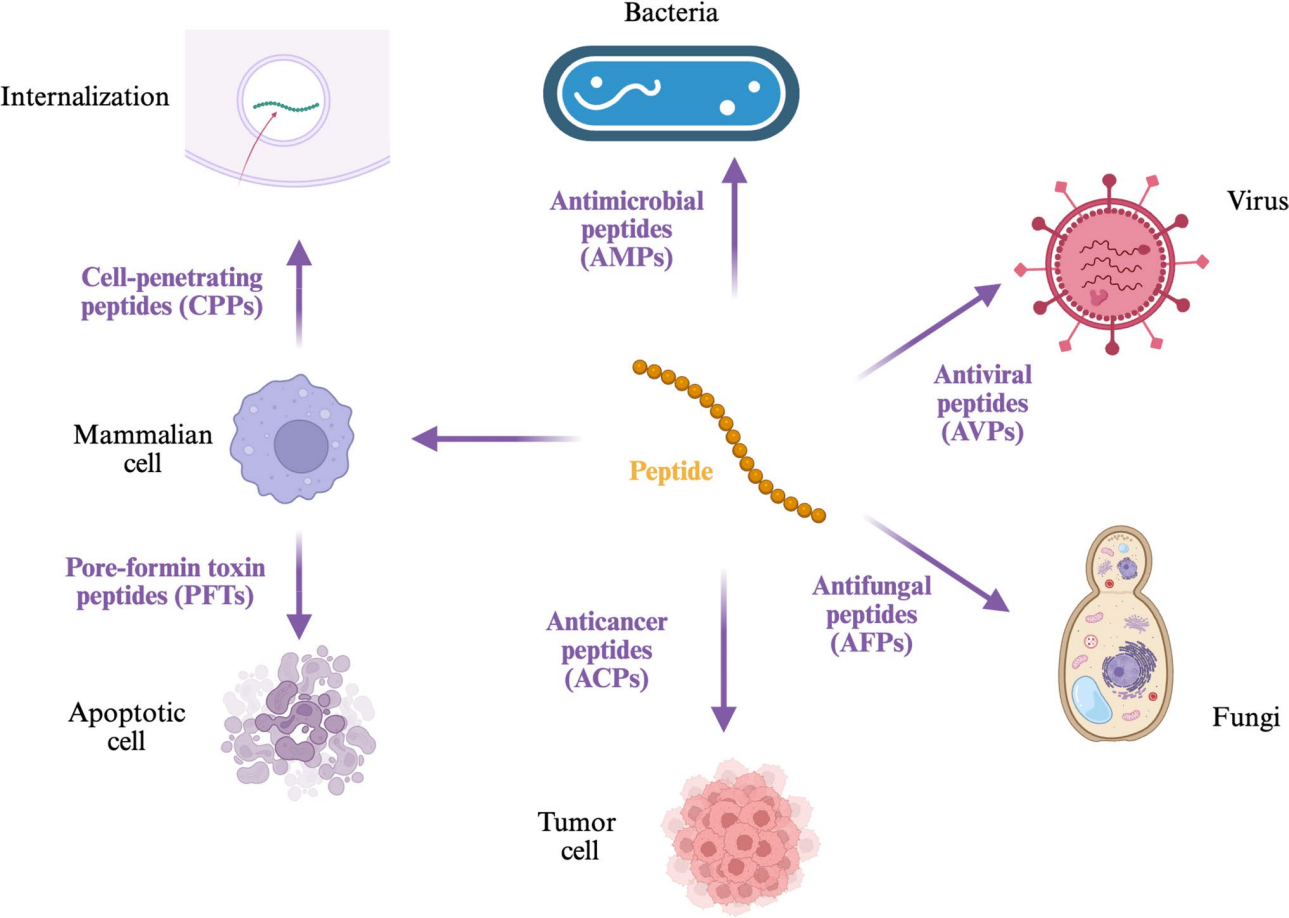
Types of membrane-active peptides

Membrane disruption is a phenomenon where the lipid bilayer’s structure is altered, either temporarily or permanently (McNeil And Steinhardt 2003). This can result from various factors including harsh environmental conditions (e.g., extreme pH levels, heat, and electric fields), exposure to detergents, actions of virulence factors produced by pathogens, or interaction with peptides. Peptides demonstrate a notable ability to interact with membranes in diverse ways. Short, and often cationic, peptides that primarily exert their biological activity through membrane interaction are referred to as *membrane active peptides* (MAPs) (Avci et al. 2018). MAPs belong to a broader category known as bioactive peptides (BPs) (Aluko 2012), which are short amino acid sequences capable of modulating physiological processes like immune response, antioxidant defense, blood pressure regulation, and appetite control (Kadam et al. 2015). Alongside proteins, BPs play vital roles in the metabolic functions of living organisms (Sánchez and Vázquez 2017). Two main groups within MAPs are recognized (Fig. 1): antimicrobial peptides (AMPs) and cell-penetrating peptides (CPPs) (Avci et al. 2018).

AMPs are typically short, cationic peptides exhibiting broad-spectrum activity against various pathogens including bacteria, viruses, fungi, and protozoa (Izadpanah And Gallo 2005). Their mechanism often involves electrostatic interactions with negatively charged components of microbial membranes, leading to membrane destabilization, pore formation, and rapid cell lysis. CPPs, while also short and cationic, interact differently; they facilitate their own internalization by inducing temporary, localized changes in the lipid bilayer, allowing translocation into the cell without causing lasting damage or compromising cellular viability (Lindgren et al. 2000). Interestingly, AMPs can utilize a mechanism similar to CPPs at lower concentrations, while exhibiting membrane leakage at higher concentrations (Rice et al. 2025).

Furthermore, other peptide groups also interact with membranes and are considered MAPs (Fig. 1). For example, anticancer peptides (ACPs) are also short BPs, generally between 5 and 50 amino acids long, that selectively exhibit cytotoxicity toward cancer cells while sparing healthy tissues (Xie et al. 2020; Zhang et al. 2023). Their cationic and amphipathic nature allows them to preferentially bind to the negatively charged membranes of tumor cells, leading to destabilization and cell death. ACPs hold significant therapeutic potential for next-generation cancer treatments (Kurrikoff et al. 2019; Zhang et al. 2023).

Antiviral peptides (AVPs) are BPs that inhibit viral infections through various mechanisms, including binding to viral envelope proteins or host cell receptors, and disrupting viral membranes via pore formation (Gao et al. 2021; Qureshi 2025). These capabilities make AVPs promising candidates for broad-spectrum antiviral therapies (Vilas Boas et al. 2019).

Antifungal peptides (AFPs) target fungal pathogens with high specificity and minimal toxicity to mammalian cells (Ciociola et al. 2016; Li et al. 2021a). Primarily, AFPs disrupt fungal cell membranes and interfere with cell wall biosynthesis, exploiting the unique composition of fungal membranes (rich in chitin, β-glucans, and mannoproteins) for selectivity. AFPs offer a strong foundation for alternatives to traditional antifungal agents, particularly given increasing drug resistance (Bondaryk et al. 2017).

Finally, pore-forming toxin peptides (PFTs) are peptides predominantly secreted by pathogenic bacteria that form transmembrane pores and compromise host cell integrity (Li et al. 2021b; Lata et al. 2022). The formation of these pores disrupts cellular ion gradients, leading to unregulated flux and potential cell lysis or apoptosis. PFTs represent important virulence factors and potential targets for therapeutic intervention (Popoff 2024).

## CPP history

Early investigations into MAPs demonstrated that certain naturally occurring peptide sequences inherently disrupt biological membranes. Among the earliest examples were melittin, the principal peptide in bee venom (Terwilliger et al. 1982), alamethicin, a voltage-dependent pore-forming peptide (Nagaraj And Balaram 1981), and magainin, an AMP isolated from amphibian skin (Zasloff et al. 1988). These peptides, extensively characterized during the 1980 s, were shown to permeabilize or destabilize lipid bilayers through various mechanisms primarily involving pore formation, rather than through non-cytotoxic membrane translocation. This conceptual distinction subsequently provided the foundation for identifying and understanding CPPs.

The first observation of cell-penetrating behavior is linked to the trans-activator of transcription (TAT) protein from human immunodeficiency virus (HIV)−1, reported in 1988. TAT demonstrated the ability to cross cellular membranes without apparent harm to the lipid bilayer, facilitating the activation of the HIV-1 promoter (Green And Loewenstein 1988; Frankel And Pabo 1988). This unexpected property was subsequently linked to a short, highly basic amino acid sequence within the TAT protein, now commonly referred to as the TAT CPP, characterized by a high concentration of lysine and arginine residues (Vivès et al. 1997).

In 1991, a similar phenomenon was observed for the Antennapedia protein from *Drosophila melanogaster*, a transcription factor crucial for regulating morphological differentiation (Joliot et al. 1991). Researchers found that its activity to cross membranes resided within the third α-helix of the protein, a segment later named penetratin (Derossit et al. 1994). Like TAT, penetratin is also rich in lysine and arginine amino acids, suggesting that electrostatic interactions with membrane phospholipids and glycosaminoglycans are important for facilitating cellular uptake.

**Fig. 2:**
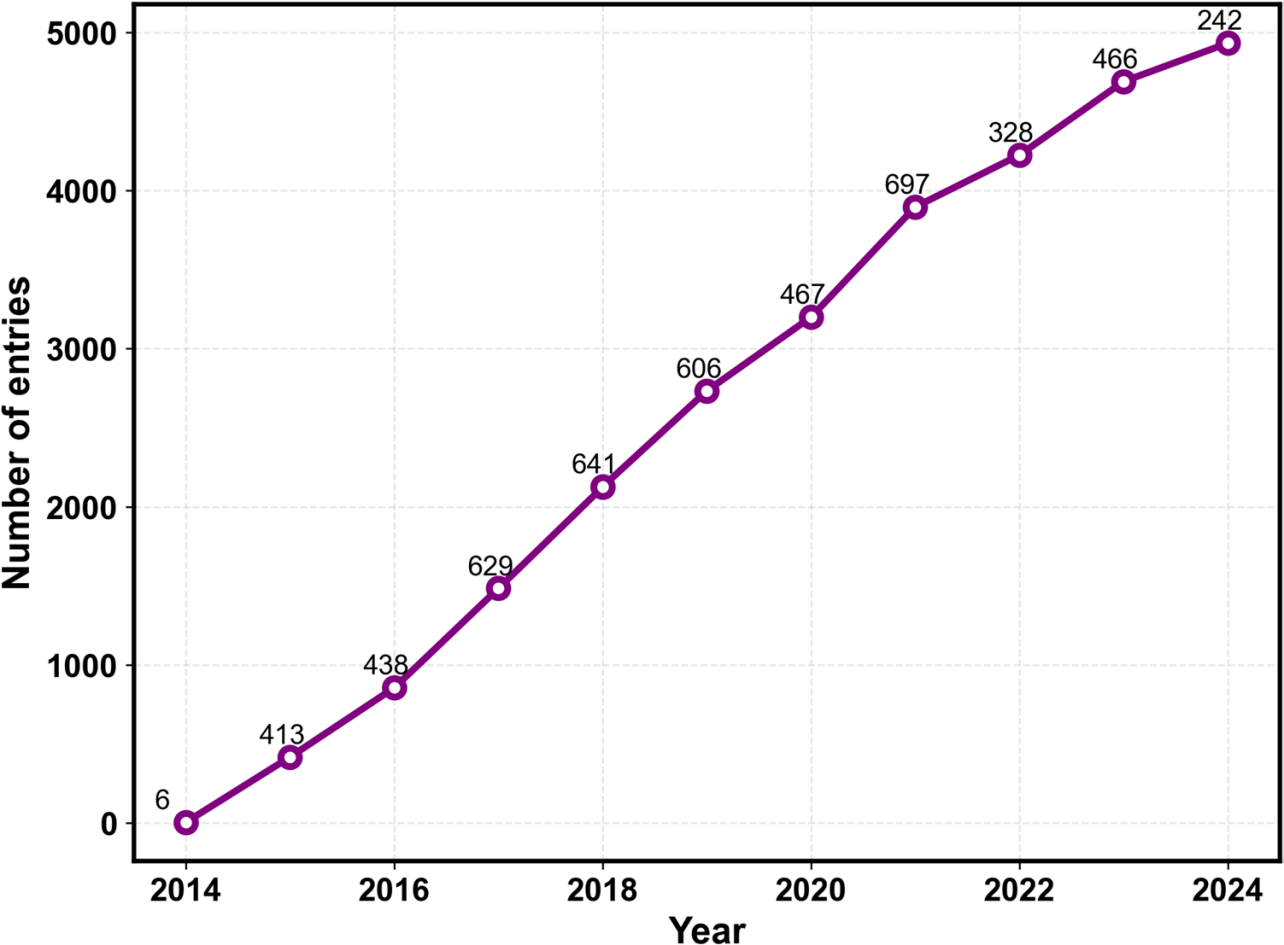
Total number of entries each year in CPPsite3.0 (Bajiya et al. 2025). The label indicates the number of new entries added to the database per year

These initial discoveries of TAT and penetratin demonstrated the potential of CPPs to cross cell membranes independently or while carrying cargo. In the subsequent years, numerous new CPPs were identified, both naturally occurring and synthetically designed, many of them based on poly-arginine designs. The field has since grown rapidly (Fig. 2), with comprehensive databases like CPPsite3.0 (Bajiya et al. 2025) now containing thousands of entries featuring diverse sequences and delivery capabilities. Rapid database growth also exacerbates a key computational issue: few experimentally confirmed non-CPPs relative to positives, which biases classifiers and complicates cross-study comparability, a theme revisited in other reviews (Ramasundaram et al. 2024).

## Classification

**Fig. 3:**
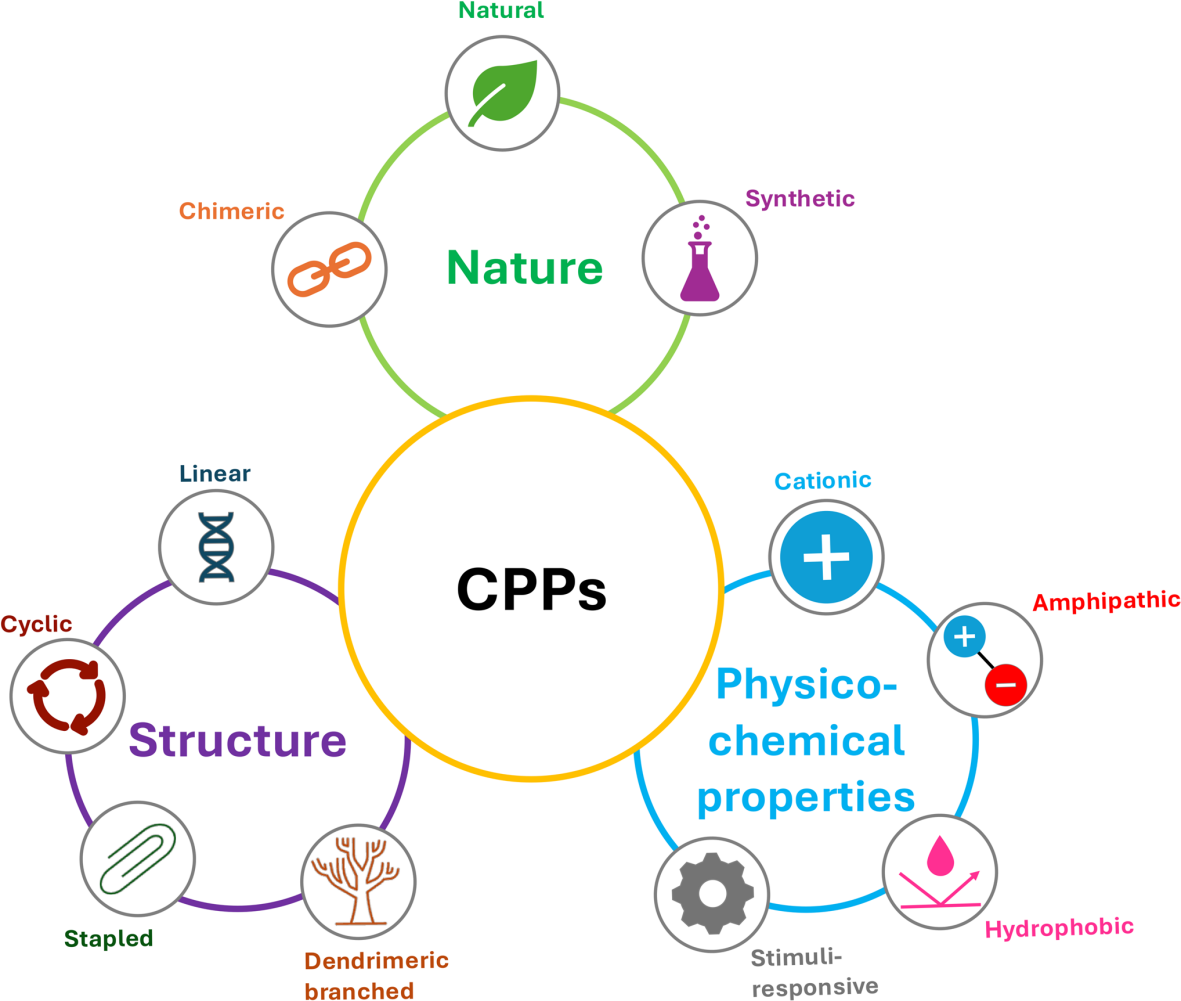
Classification of CPPs according to their physicochemical properties, nature, and structural characteristics

Many naturally occurring peptides and proteins have evolved specialized mechanisms that enable them to interact with, and in some cases traverse, biological membranes. Drawing inspiration from these natural processes, researchers have developed CPPs that utilize comparable physicochemical strategies. CPPs can be classified according to various criteria, including their origin, structural features, and physicochemical characteristics (Fig. 3). Some CPPs are directly derived from native protein sequences, whereas others are rationally engineered to respond to environmental stimuli, such as electric potential, temperature fluctuations, or pH changes, that can profoundly influence membrane organization and peptide dynamics.

Based on their physicochemical properties, CPPs are generally categorized into four main types: cationic, amphipathic, hydrophobic, and stimuli-responsive CPPs. Cationic CPPs (such as polyarginine, Arg9 (Futaki et al. 2001), TAT, or penetratin) often contain a high proportion of positively charged amino acids like arginine and lysine, which facilitate interactions with the negatively charged surfaces of cell membranes. The positive charge density is frequently utilized to promote cellular entry (Milletti 2012). However, other amino acid characteristics can also play a role in uptake, as is the case for penetratin, where a mutated tryptophan drastically reduces cellular uptake (Prochiantz 1996).

Amphipathic CPPs, such as model amphipathic peptide, MAP (Steiner et al. 1991; Silva et al. 2022), transportan 10, TP10 (Yandek et al. 2007), or Pep-1 (Morris et al. 2001), possess hydrophilic and hydrophobic parts, enabling them to interact with both the surrounding aqueous environment and the lipid components of cell membranes. This group can be further subdivided based on their structure into primary, secondary α-helical, secondary β-sheet, and proline-rich subclasses. Primary amphipathic peptides have a naturally distributed arrangement of hydrophilic and hydrophobic amino acids. Secondary amphipathic CPPs are unstructured in solution but adopt an α-helix or β-sheet conformation when interacting with a membrane (Rádis-Baptista et al. 2017). The third group, proline-rich CPPs, contains a high proportion of prolines, which can lead to the formation of a unique left-handed helical polyproline helix (PPII), with 3.0 residues per turn (instead of the 3.6 residues per turn in the right-handed conventional α-helix) (Adzhubei et al. 2013). PPII helices are known for their ability to interact with proteins and nucleic acids, often acting as recognition sites and allowing peptide interactions (Hicks And Hsu 2004; Cubellis et al. 2005; Mansiaux et al. 2011).

Hydrophobic CPPs primarily consist of nonpolar amino acid residues and have been less extensively studied compared to the other categories. They rely on hydrophobic interactions to cross cell membranes. While relatively understudied, they have significant examples such as Kaposi fibroblast growth factor (K-FGF) (Lin et al. 1995) or translocating peptide 2 (TP2) (Milletti 2012). These sequences were found to directly enter cells, bypassing the usual cellular pathways, being directly available in the cytosol and reducing the risk of being trapped within endosomes (Marks et al. 2011).

Stimuli-responsive CPPs exhibit membrane activity predominantly under acidic conditions, allowing their selective activation within the tumor microenvironment. Representative examples include pH-(low)-insertion peptide (Hunt et al. 1997; An et al. 2010) and the histidine-rich synthetic peptide LAH4-L1 (Wolf et al. 2017). This pH-dependent behavior enhances tumor specificity and facilitates the localized release of therapeutic cargo at the pathological site (Lee et al. 2017).

Anionic CPPs do not constitute a distinct class on their own (Ashrafuzzaman 2018); however, SAP10 (Tsai et al. 2018), LE10 (Antunes et al. 2013), and p28 (Yamada et al. 2013) are representative examples of this type of CPP.

CPPs are also classified and categorized by their origin or design. Here, three groups are included: natural CPPs, derived from existing proteins; chimeric CPPs, created by combining segments from different sources; and synthetic CPPs, completely designed from scratch. TAT and penetratin are examples of natural CPPs, while transportan and Pep-1 are considered chimeric or synthetic.

Despite their potential, CPPs have limitations such as instability in biological environments, possible toxicity, and challenges with crossing certain types of cell membranes. Researchers are actively working to address these drawbacks, for instance, by modifying the structure of CPPs to improve stability, enhance delivery efficiency, and reduce adverse effects (Gori et al. 2023). These modifications have given rise to another classification system based on structural design.

While most CPPs have a linear arrangement, alternative designs like cyclic, stapled, and dendrimeric structures have been developed to increase resistance to enzymatic breakdown (proteolysis) (Qian et al. 2016; Fang et al. 2022). Cyclic peptides often show improved penetration capabilities compared to their linear counterparts, along with reduced toxicity and greater stability, which make cyclic CPPs a promising new tool (Qian et al. 2012; Dougherty et al. 2019; Sajid et al. 2021). Similarly, stapled CPPs exhibit increased resistance to degradation and enhanced cell permeability (Tian et al. 2017). In addition, branched dendritic peptides, resembling a tree-like structure, can also improve cellular uptake and overall stability (Breger et al. 2017).

It is important to note that the classification of CPPs is not always rigid; some peptides may display characteristics of multiple groups, and their behavior can be influenced by factors such as concentration, experimental conditions, and the nature of the cargo being transported. Understanding these classifications is essential for predicting how a peptide will interact with cell membranes and its overall effectiveness in drug delivery. Beyond taxonomy, peptide chemistry also predicts entry physics: Arg-rich CPPs tend to utilize electrostatically assisted, defect-, or pore-mediated pathways, whereas hydrophobic CPPs (e.g., TP2) more often insert with limited membrane disruption. This distinction should guide the selection of simulation methods, for example, utilizing CompEL/aSMD for pore-prone systems versus CG → AA back-mapping for insertion-prone ones (J. Bond And Khalid 2012; Ouyang et al. 2022).

## Mechanism of entry

**Fig. 4:**
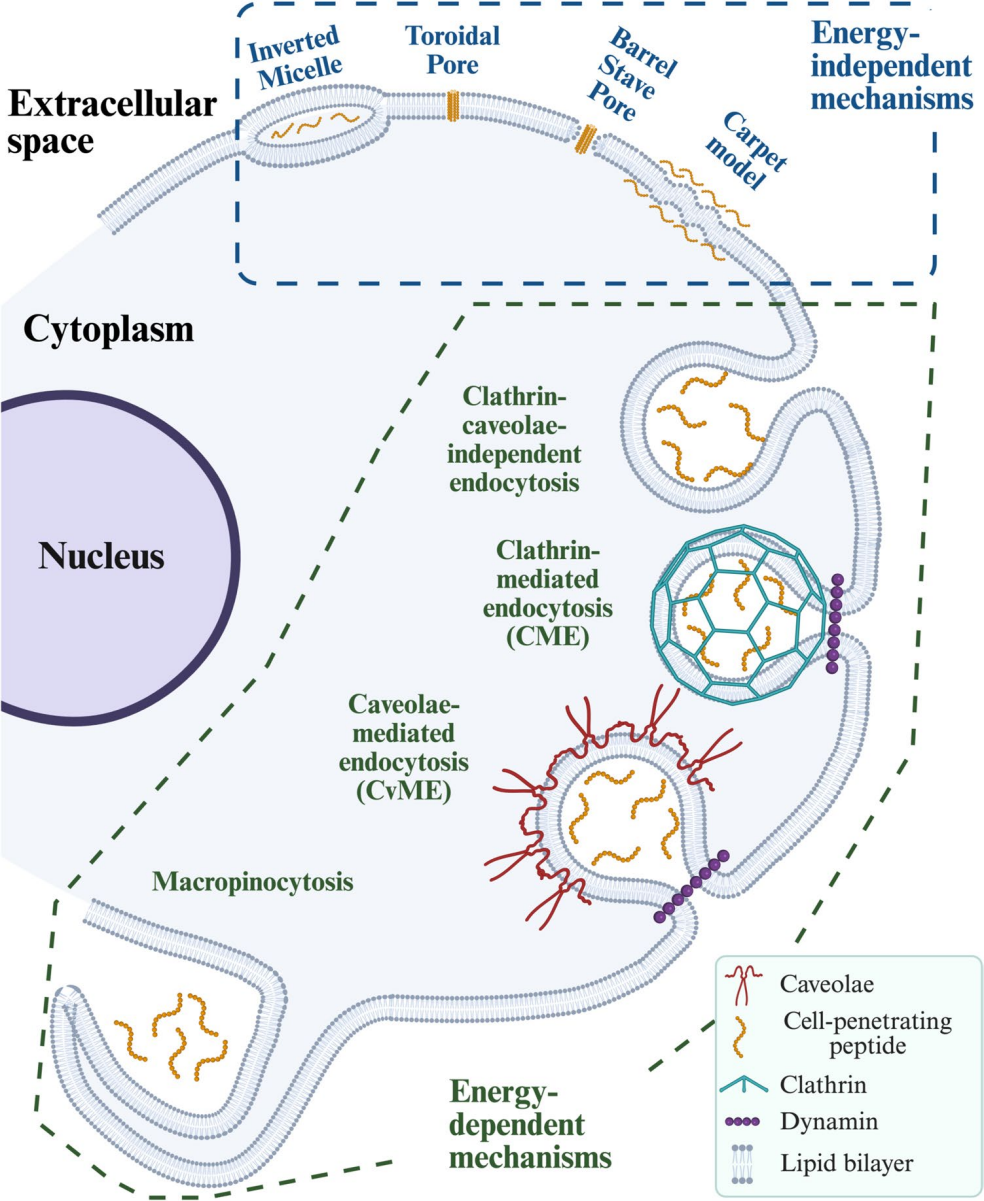
Energy-independent and energy-dependent mechanisms of CPPs’ entry into the cell. Created in https://BioRender.com

The mechanism by which CPPs cross mammalian cell membranes has remained a subject of inquiry and debate, particularly regarding the prevalence and specifics of different uptake mechanisms (Datta 2025). Several pathways have been proposed to explain how these peptides are able to enter cells, which can be classified into energy-independent and energy-dependent mechanisms (Fig. 4). Energy-independent mechanisms entail direct translocation across the plasma membrane, whereas energy-dependent processes involve vesicle formation and endocytosis (Ruseska And Zimmer 2020).

### Energy-independent pathways

Direct penetration mechanisms represent energy-independent pathways of cellular uptake, with several models proposed to explain them, including the inverted micelle, carpet, membrane thinning, and pore formation (e.g., barrel-stave and toroidal pores) models. The initial phase of this process involves electrostatic interactions between the peptide and the plasma membrane, which modify the supramolecular organization of lipids.

In the inverted micelle model, these electrostatic forces induce local changes in membrane curvature, such as invaginations (Alves et al. 2010). Such curvature alterations can promote the generation of inverted micelles that encapsulate the peptide. Subsequent micelle destabilization enables the release of the peptide-cargo complex into the cytoplasm. This mechanism has been described for TAT and oligoarginine peptides interacting with negatively charged vesicles (Swiecicki et al. 2014).

Alternatively, peptide-induced membrane perturbation can result in translocation through pore-formation mechanisms, which include the barrel-stave and toroidal models. In the barrel-stave model, peptide monomers aggregate upon membrane contact to form bundles with central aqueous channels, allowing additional peptides to traverse the membrane (Copolovici et al. 2014). In the toroidal model, the peptides adopt α-helical conformations upon membrane binding, bending the bilayer and generating pores that involve the lipid headgroups (Bechara And Sagan 2013; Islam et al. 2018). Oligoarginines, TP10, and TAT are thought to translocate through such pore-forming processes (Mishra et al. 2008, 2011; Islam et al. 2014).

In the carpet model, peptides orient parallel to the membrane surface, where their cationic residues associate with negatively charged phospholipid headgroups while their hydrophobic segments interact with the lipid bilayer core. This configuration, first proposed by Pouny et al. (1992), resembles a “carpet” covering the membrane surface. Once a critical threshold concentration is reached, this arrangement disrupts local lipid packing, leading to membrane destabilization and promoting peptide internalization.

Taken together, simulations and experiments converge on a chemistry-dependent dichotomy: Arg-rich CPPs (e.g., Arg9) typically require or strongly benefit from transient, water-filled defects to maintain hydration and headgroup contacts during translocation, whereas hydrophobic CPPs (e.g., TP2) can embed and traverse with minimal pore stabilization. This mechanistic split offers a design heuristic for tuning uptake while managing membrane perturbation (Ouyang et al. 2022; Castelletto et al. 2024).

### Energy-dependent pathways

Endocytosis represents an energy-dependent internalization pathway that relies on vesicle formation to transport peptides into the cell. In this process, the positively charged residues of many peptides mediate their initial electrostatic interactions with negatively charged components at the cell surface, facilitating the uptake of the peptide-cargo complex within vesicular compartments. Multiple endocytic routes have been implicated in this process, including macropinocytosis, clathrin-mediated endocytosis (CME), caveolae-mediated endocytosis (CvME), and clathrin- and caveolae-independent pathways (Conner And Schmid 2003).

Macropinocytosis is a rapid, “lipid raft”-dependent, and receptor-independent mechanism of endocytosis (Wadia et al., 2004). It is typically activated by growth factors or other stimuli that promote actin-driven membrane ruffling in various cell types. Unlike receptor-mediated internalization, macropinocytosis does not rely on specific ligand-receptor interactions. Instead, it involves the formation of large, actin-supported membrane protrusions that fold back and fuse with the plasma membrane, generating vesicles known as macropinosomes. This process enhances fluid-phase uptake and enables non-selective internalization of extracellular material, including peptides. Arginine-rich peptides such as TAT frequently exploit this pathway, particularly at elevated concentrations.

CME is among the most extensively characterized and common pathways for cellular uptake mechanisms (Ruseska And Zimmer 2020). It is a receptor-dependent and clathrin- and dynamin-mediated process that operates in nearly all mammalian cells (Xiang et al. 2012). CME begins when a ligand, such as a peptide or nutrient, binds to its specific surface receptor, initiating the recruitment of clathrin to form a polyhedral lattice on the cytosolic side of the membrane. This leads to the development of clathrin-coated pits, which progressively invaginate to form dome-shaped structures. Dynamin, a GTPase, mediates the final membrane scission, releasing clathrin-coated vesicles (CCVs) into the cytoplasm. These vesicles are subsequently uncoated and delivered to early endosomes, which can mature into late endosomes and eventually fuse with lysosomes (Schmidt et al. 2010). Arginine-rich peptides have been shown to utilize CME as one of their internalization routes.

Caveolae-mediated endocytosis (CvME) is a clathrin-independent but dynamin-dependent pathway that relies on caveolae (small, flask-shaped membrane invaginations approximately 50–100 nm in diameter) for internalization (Fittipaldi et al. 2003). These structures are enriched in cholesterol, sphingolipids, and the protein caveolin and are particularly abundant in endothelial and adipose tissues. Caveolae are considered a specialized type of lipid domain, serving as dynamic microdomains involved in signaling and endocytosis. In the context of peptide uptake, CvME provides an alternative entry route that circumvents lysosomal degradation, thereby enhancing intracellular delivery efficiency.

Finally, clathrin- and caveolae-independent endocytosis encompasses a set of less well-defined pathways that operate independently of clathrin and caveolin and may be either dynamin-dependent or -independent. These include the CLIC/GEEC pathway (Kirkham et al. 2005), the Arf6-dependent mechanism (D’Souza-Schorey And Chavrier 2006), and flotillin-mediated endocytosis (FME) (Glebov et al. 2006).

The mechanism of cellular uptake utilized by a peptide depends on several variables, including the physicochemical properties and concentration of the peptide, the nature of its cargo, and the composition of the target membrane. For example, penetratin, TAT, and Arg9 predominantly internalize via endocytic pathways such as macropinocytosis, CME, and CvME (Zakany et al. 2023). Although endocytosis is thought to account for most peptide internalization events, direct translocation can occur at elevated peptide concentrations (Palm-Apergi et al. 2012). Interestingly, penetratin represents an exception: at low concentrations, it crosses the plasma membrane through an energy-independent process, whereas at higher concentrations, it relies on endocytic uptake (Pae et al. 2014). Thus, both the uptake mechanism and its relative contribution can vary depending on the specific peptide (Zakany et al. 2023). Amphipathic CPPs tend to internalize preferentially through more dynamic membrane regions, while arginine-rich CPPs largely depend on interactions with membrane-associated proteins (Futaki et al. 2007; Åmand et al. 2012; Takeuchi and Futaki 2016; Almeida et al. 2019). An often-overlooked factor in these processes is the transmembrane potential (Δ*Ψ*), which lowers the energetic threshold for transient pore formation and can tilt the balance between direct translocation and endocytic uptake. Multiple studies support a facilitating role for Δ*Ψ*, suggesting it is a significant beneficial factor, even if not strictly necessary for translocation (Rothbard et al. 2004; Zhang et al. 2009; Via et al. 2018; Gao et al. 2019a).

## Computational techniques

Experimental methodologies such as spectroscopy, microscopy, and biochemical assays have been instrumented in elucidating the structural properties, behavior, and membrane interactions of CPPs (Holm et al. 2006; Zorko And Langel 2022). However, these techniques often face inherent limitations in resolving atomic-scale details or capturing the dynamic and transient events that govern CPP-membrane interactions. Computational approaches address these challenges by providing atomic insight into peptide structure, dynamics, and membrane interaction. Molecular simulations, in particular, enable the rational design of CPPs with enhanced efficiency and selectivity, thereby supporting the development of more effective intracellular delivery systems. In this context, computational modeling serves as a valuable complement to experimental work, offering predictive frameworks that can guide hypothesis-driven research and design in CPP-based therapeutics.

In this review, we focus on molecular dynamics (MD) simulations (Alder And Wainwright 1957; Rahman 1964) as a primary computational tool to examine how CPPs adopt specific conformations, interact with lipid bilayers, and respond to environmental or sequence-dependent variations. MD simulations operate by numerically solving Newton’s equations of motion, utilizing predefined force fields (sets of equations and parameters) to compute atomic and molecular trajectories over time. The resulting simulation trajectory provides a detailed, time-resolved description of the motion of all atoms within the system (Karplus And Petsko 1990).

To critically synthesize prior work, our analysis explicitly considers three cross-cutting constraints: first, the biology-simulation timescale gap, which contrasts biological events occurring over milliseconds or minutes with the microsecond-scale trajectories common to MD; second, the necessity of membrane realism, incorporating cholesterol, anionic lipids, and leaflet asymmetry, as these factors significantly modulate entry pathways; and third, the data imbalance in computational screening, particularly the paucity of validated non-CPPs that inflates false-positive rates in machine learning predictors (Ouyang et al. 2022; Ramasundaram et al. 2024).

### Existing methods

**Fig. 5:**
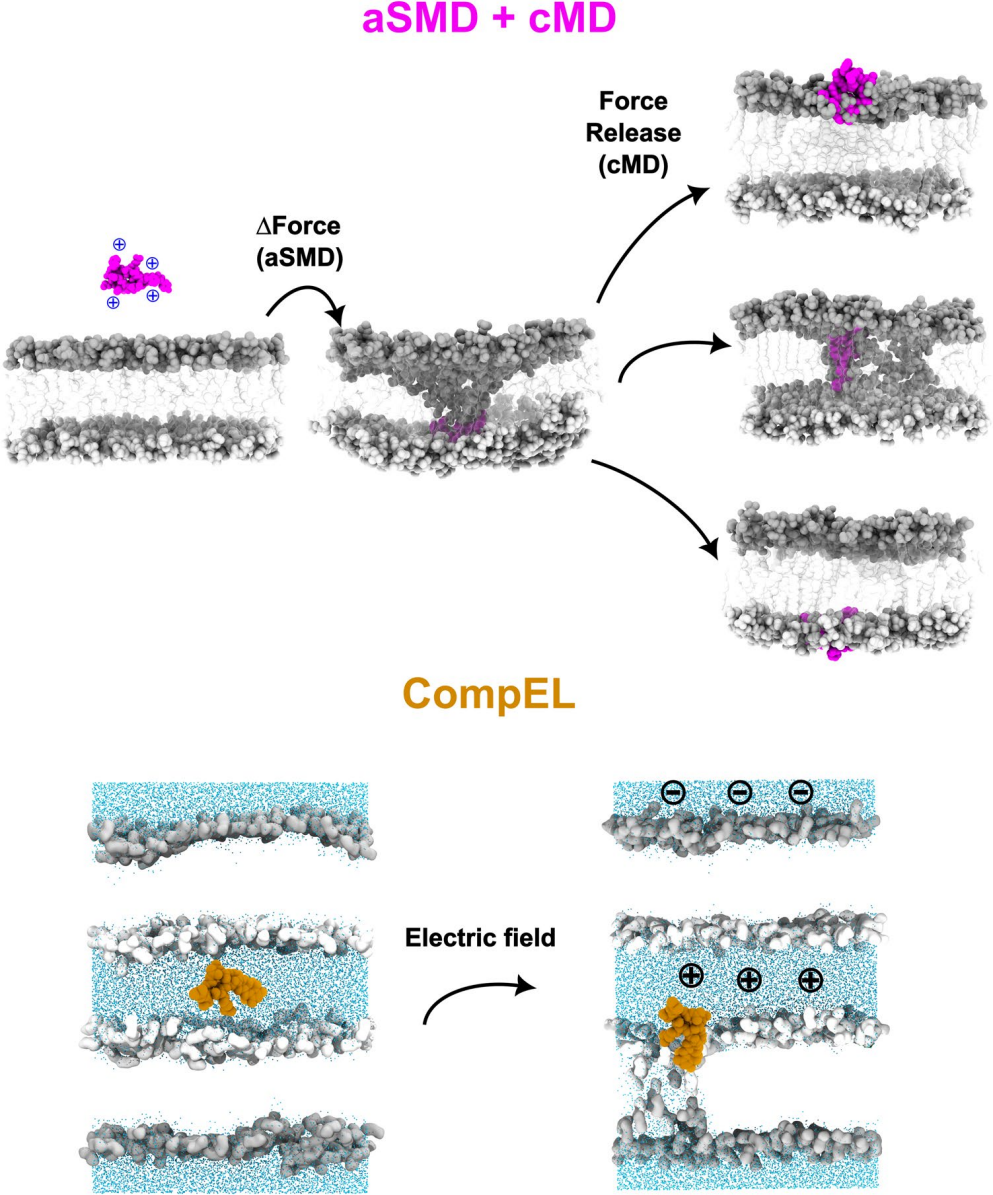
Summary of the computational techniques used by our group in CPP translocation study: adaptive steered molecular dynamics in combination with conventional molecular dynamics (aSMD + cMD, upper part) and computational electrophysiology (CompEL, lower part)

Within the CPP research field, MD simulations are particularly valuable for investigating peptide-membrane interactions. Nevertheless, direct observation of translocation events remains challenging because the timescales involved (seconds to minutes (Yesylevskyy et al. 2009)) greatly exceed those accessible to conventional molecular dynamics (cMD), which is typically currently limited to the microsecond range due to computational constraints. To overcome these limitations, a variety of strategies, most notably enhanced sampling techniques, have been developed to enable the detailed study of CPP interactions and translocation mechanisms (Fig. 5). However, the explanation of all enhanced sampling molecular dynamics methods is out of the scope of this review, and readers are encouraged to refer to available excellent reviews (Bernardi et al. 2015; Hénin et al. 2022).

Instead, we limited this part of the review to mention the main computational techniques used in CPP study. First, steered molecular dynamics (SMD) is one of the most widely applied approaches. This method is particularly useful for accelerating the translocation of peptides across membranes and for computing the associated free energy profile or potential of mean force (PMF) (Park And Schulten 2004; Yesylevskyy et al. 2009). Nevertheless, when the reaction coordinate involves a long or highly curved path, conventional SMD may suffer from significant deviations and increased statistical noise, so the adaptive steered molecular dynamics (aSMD) (Ozer et al. 2010; Gimenez-Dejoz And Numata 2022) was introduced as a refined variant designed for systems where the steered group follows complex, non-linear trajectories. Another prominent approach is metadynamics (MetD), which reconstructs the system’s free energy surface using a limited number of collective variables (CVs) (Laio And Parrinello 2002; Barducci et al. 2011), allowing sampling of high-energy or rarely visited regions of the free energy landscape. Similarly, umbrella sampling (US) achieves enhanced exploration by dividing the reaction coordinate (here, the peptide’s translocation path) into overlapping “windows” while ensuring efficient sampling of otherwise unfavorable configurations (Torrie And Valleau 1977). This method also facilitates precise PMF determination across the entire reaction coordinate (Yesylevskyy et al. 2009; Alaybeyoglu et al. 2016). In replica exchange molecular dynamics (REMD), multiple replicas of the system are simulated in parallel under different thermodynamic conditions, such as varying temperatures. Periodic exchanges between replicas enable the system to overcome high-energy barriers and improve configurational sampling (Sugita And Okamoto 1999). Although REMD is not typically used alone for PMF computation, it can be combined with US (RE + US) to enhance sampling efficiency and convergence (Kabelka et al. 2021). Moreover, the weighted ensemble (WE) (Zuckerman And Chong 2017; Choe 2021) method divides the configurational space into discrete bins and runs multiple parallel trajectories, which enables efficient exploration of rare but biologically relevant transitions, providing detailed insights into the kinetics and mechanisms of CPP translocation.

Across enhanced sampling approaches, relative peptide ranking by barrier is often consistent when collective variables are well-posed; however, absolute PMFs diverge systematically (for instance, non-equilibrium aSMD typically exceeds US) due to work-distribution tails and whether the coordinate spans full-bilayer versus half-bilayer passage (Noh And Notman 2020; Paloncýová et al. 2023; Dutta et al. 2023). We recommend explicit reporting of membrane composition (cholesterol/anionic lipid content), ionic strength, pulling speeds, CV definitions, and PMF reference states to improve cross-study comparability (Torrie And Valleau 1977; Ozer et al. 2010; Catalina-Hernandez et al. 2025c).

In addition to enhanced sampling methods, several alternative MD strategies have been developed that can facilitate or accelerate peptide translocation across membranes. One such approach is high-temperature molecular dynamics (HT-MD), in which simulations are conducted at elevated temperatures to increase molecular motion, accelerate kinetic events, and promote greater membrane perturbation (Wang et al. 2016). Similarly, membrane-tension molecular dynamics (MT-MD) involves applying lateral stress to the lipid bilayer to enhance its permeability and induce structural disruption, thereby facilitating peptide insertion or passage (He et al. 2015; Rice et al. 2025).

Another widely employed approach is coarse-grained molecular dynamics (CG-MD), which simplifies the system by grouping multiple atoms into larger beads, thus reducing resolution but achieving significantly longer timescales (Hu et al. 2014; Marrink et al. 2023). However, spontaneous peptide translocation is not always observed within accessible simulation times. To overcome this limitation, CG-MD is frequently combined with other techniques such as US or SMD to further increase sampling efficiency and improve representation of translocation events (Suresh Patel And Ytreberg 2017; Sieradzan And Jakubowski 2017).

A different class of modeling strategies involves the use of implicit membrane models (IMM), which approximate the lipid bilayer and solvent environment through continuum representations embedded within the system’s solvation-free energy function. This approach enables rapid equilibration and broad exploration of configurational space due to the absence of explicit solvent and lipid molecules. A notable example is IMM1, developed by Lazaridis et al., which allows the estimation of minimum-energy pathways and transition-state energies during CPP translocation (Lazaridis et al. 2014).

Despite their utility, each of these techniques presents inherent limitations (Table 1). Methods such as SMD and US rely on externally applied biases that, while useful for PMF determination, may distort natural dynamics. CG-MD sacrifices atomic-level detail, and spontaneous insertion or translocation cannot always be observed. IMM1 is limited by its applicability to α-helical peptides and inability to explicitly represent membrane-peptide interactions. Likewise, HT-MD assumes that force fields remain valid at elevated temperatures, whereas MT-MD employs non-physiological membrane tension. Finally, approaches such as the WE method are highly dependent on the accurate definition of reaction coordinates. In addition, most of these techniques also require significant computational resources and specialized expertise, particularly in parameter selection and reaction coordinate design, which limits their accessibility to non-expert users.

**Table 1:** Summary of the characteristics of the computational techniques used in CPP study

Technique	Resolution	PMF calculation^1^	Peptide translocation	Entry-level	Computational requirements	References
cMD	Atomic	No	No	Yes	Low	Ivánczi et al. (2023)
SMD	Atomic	Yes	Yes	No	Moderate	Yesylevskyy et al. (2009); Alaybeyoglu et al. (2016)
aSMD	Atomic	Yes	Yes	No	Intensive	Gimenez-Dejoz and Numata (2022)
MetD	Atomic	Yes	Yes	No	Moderate	Cao et al. (2020)
US	Atomic	Yes	Yes	No	Intensive	Grasso et al. (2015); V. Mathath et al. (2026)
HT-MD	Atomic	No	Yes	Yes	Moderate	Wang et al. (2016)
RE + US	Atomic	Yes	Yes	No	Intensive	Kabelka et al. (2021)
WE	Atomic	Yes	Yes	No	Intensive	Choe (2021, 2023, 2024)
aSMD + cMD	Atomic	Yes	Yes	No	Intensive	Catalina-Hernandez et al. (2025b, c)
CompEL	Atomic	No	Yes	Yes	Moderate	Catalina-Hernandez et al. (2025a)
MT-MD	Atomic	No	Yes	No	Moderate	He et al. (2015); Rice et al. (2025)
CG	Beads	No	Not efficiently	No	Low	Kawamoto et al. (2011, 2012); Hu et al. (2014 ); Suresh Patel and Ytreberg (2017); Sieradzan and Jakubowski (2017)
IMM	Atomic, but without membrane and waters	Yes	No	No	Low	Lazaridis (2005); Lazaridis et al. (2014); Nepal et al. (2018)

^1^The possibility of calculating the PMF of peptide translocation across the membrane. It is important to note that even though some techniques do not allow for PMF calculation, they can be combined with other techniques that allow it

### New presented methods

To address the methodological and computational limitations associated with cMD techniques, our laboratory has presented specialized simulation strategies designed to provide atomistic insight into peptide-membrane interactions and translocation processes under more physiologically relevant conditions. These approaches, namely the combined adaptive steered molecular dynamics with conventional molecular dynamics (aSMD + cMD) framework (Catalina-Hernandez et al. 2025b, c) and the computational electrophysiology (CompEL) method (Catalina-Hernandez et al. 2025a), were specifically conceived to overcome the constraints of traditional and enhanced sampling techniques while maintaining atomic-level accuracy and mechanistic interpretability.

First, the aSMD + cMD protocol enables both quantitative free-energy estimation (through PMF calculation) and qualitative assessment of membrane perturbation following peptide translocation. In this hybrid workflow, aSMD is employed to actively steer the peptide across the lipid bilayer, allowing calculation of the PMF associated with membrane translocation. Subsequent cMD simulation then captures the relaxation and reorganization of the peptide-membrane system, providing detailed structural and dynamical information at atomic resolution.

This technique makes it possible to monitor every major stage involved in peptide translocation across a lipid bilayer. Peptide-membrane interaction begins with an initial association with the outer region of the upper part of the bilayer leaflet (hereafter referred to as the upper leaflet), a step commonly described as partitioning. At this stage, positively charged amino acids within the peptide sequence, such as arginine, lysine, and histidine, promote binding to the negatively charged phospholipid headgroups. As a result, electrostatic forces and hydrogen bonding dominate the early peptide-membrane contacts.

Following this interaction, the peptide progresses through the headgroup region toward the hydrophobic center of the membrane, in a step known as insertion. This phase is driven primarily by hydrophobic interactions between nonpolar peptide residues (e.g., valine, leucine, and isoleucine) and the lipid acyl chains. Despite penetration into the membrane, charged residues often remain associated with the polar headgroups, as these interactions are energetically difficult to disrupt. Consequently, lipid headgroups can be pulled inward along with the peptide, marking the onset of peptide-induced membrane perturbation (Bennett et al. 2024).

If the disturbance extends sufficiently into the bilayer, the peptide and associated headgroups may reach the polar region of the opposite leaflet (hereafter termed the lower leaflet), allowing interactions between headgroups from both sides of the membrane. When water molecules infiltrate this defect, a transmembrane pore may form if water molecules permeate the disturbance (MacCallum et al. 2008; Sun et al. 2014). Certain peptides, including Arg9, rely on such pore formation to traverse the bilayer (MacCallum et al. 2008; Irudayam And Berkowitz 2012; Klug et al. 2024; Maia et al. 2025). In the case of Arg9, this requirement arises from its highly cationic composition, which disfavors contact with the hydrophobic core and necessitates continuous interaction with either lipid headgroups or water molecules.

In the final stage, the peptide advances further into the membrane, potentially breaking its interactions with headgroups from the upper leaflet, which may then relax back to their original positions, sometimes resealing the membrane defect of closing the pore (Sun et al. 2014). Ultimately, the peptide becomes stabilized within the lower leaflet, completing the translocation process.

Although powerful, this approach entails substantial computational cost: the aSMD phase consists of 8 steps, with 5 ns per step, and with 25 replicas, followed by 3 replicas of 100 ns in the cMD part, resulting in a total of approximately 1.3 µs per system. Additionally, the method is technically demanding, as it requires explicit definition of a reaction coordinate; it is a linear process and is complicated to run parallel simulations, and it involves a steep learning curve.

Moreover, it is well established that aSMD often produces higher PMF estimates compared with techniques such as US, largely due to the non-equilibrium nature of the pulling process and the limited sampling of low-work trajectories necessary for the accurate application of Jarzynski’s relation (Jarzynski 1997; Park et al. 2003). As discussed in our aSMD + cMD article (Catalina-Hernandez et al. 2025c), part of this discrepancy also reflects differences in the chosen force field and in the definition of the translocation coordinate, specifically whether the PMF encompasses the full bilayer thickness. In our case, the calculated barrier corresponds to complete membrane translocation, which partly accounts for the relatively large energy values obtained. Recognizing that aSMD alone cannot always provide fully converged free-energy landscapes for such complex systems, we subsequently implemented an alternative, complementary approach based on computational electrophysiology to explore peptide-membrane dynamics under more biologically realistic conditions.

The CompEL framework likewise preserves atomic resolution and allows direct visualization of peptide translocation events. Unlike aSMD, CompEL does not impose an external steering force; instead, it induces transient pore formation and membrane disruption through the establishment of a Δ*Ψ*, thereby more closely mimicking physiological asymmetries. In biological systems, such asymmetries, arising from gradients in mass, tension, or electrical potential, are known to promote peptide penetration and transient pore formation (Matsuzaki et al. 1995; Rice et al. 2025). Numerous experimental and computational studies have shown that a Δ*Ψ* facilitates and, in some cases, triggers CPP translocation (X. Gao et al. 2019a, b; J. Lin & Alexander-Katz 2013; Moghal et al. 2019; Rothbard et al. 2004; Trofimenko et al. 2021; Via et al. 2018; X. Zhang et al. 2009), even though others concluded that it is not completely necessary, but it does favor translocation (Klug et al. 2024). In fact, a Δ*Ψ* is thought to trigger CPP translocation in cells (Rothbard et al. 2004; Zhang et al. 2009) by forming transient pores (X. Gao et al. 2019a, b; Herce et al. 2009; Herce & Garcia 2007; J. Lin & Alexander-Katz 2013; Trofimenko et al. 2021; Via et al. 2018), as demonstrated in both experimental and computational studies. Hence, CompEL captures this behavior while achieving simulation timescales comparable to those of biological processes (Kutzner).

In contrast to the aSMD + cMD approach, the CompEL method does not allow direct observation of the complete peptide translocation pathway, since membrane pores arise from the charge imbalance (∆*Q*) imposed across the bilayer. Nevertheless, peptides can exploit this electrically induced defeat to associate with the membrane and subsequently insert into the lipid bilayer, achieving insertion of the peptide in the bilayer. Under these conditions, peptides may also interact with the headgroups in the lower leaflet, thereby achieving full translocation, a process that can be followed by pore closure (Matsuzaki et al. 1995; Rice et al. 2025). This behavior highlights a key distinction between CPPs and AMPs. CPPs are typically able to access the lower leaflet and promote pore closure, resulting in transient and highly unstable membrane defects. In contrast, AMPs tend to remain embedded within the bilayer, often stabilizing pores that persist over time and ultimately compromise membrane integrity (Alimohamadi et al. 2023; Anahid et al. 2025).

From a practical point, CompEL is significantly more accessible than many enhanced sampling methods. System setup is straightforward, as no reaction coordinate needs to be predefined, and the initial configuration can be generated rapidly. Moreover, the method also supports multi-peptide simulations, allowing investigation of cooperative effects in complex membrane environments. In addition, because individual replicas are fully independent, CompEL simulations can be easily parallelized across multiple computational nodes or machines. Nonetheless, CompEL does not directly yield PMF profiles and thus must be combined with complementary techniques (e.g., aSMD or US) when quantitative free-energy information is required.

It is important to note that distinguishing between peptide insertion and full translocation may, in part, reflect the finite timescales accessible to atomistic MD. Longer simulations could potentially reveal additional events beyond those captured in our study (Catalina-Hernandez et al. 2025a). Nonetheless, in our work, TP2 simulations were extended up to 1 µs and no further translocation events were observed, suggesting that the system had reached equilibrium. Still, future work could apply enhanced sampling methods such as US or MetD to more rigorously quantify energetic barriers and refine the separation between inserted and translocated states. Despite these limitations, our current simulations already capture mechanistically distinct regimes corresponding to stable pore association versus full membrane crossing, thereby advancing understanding of peptide-membrane interactions.

Collectively, the two approaches introduced by our lab, aSMD + cMD and CompEL, expand the methodological landscape for investigating peptide-mediated membrane disruption and translocation. Each provides unique capabilities not attainable with existing simulation strategies. The aSMD + cMD protocol enables quantitative estimation of the PMF, while also allowing for atomic-level detail of peptide-membrane interactions following peptide translocation; however, this comes at the cost of substantial computational demand. Conversely, CompEL does not provide direct PMF calculation but offers a computationally efficient, more physiologically relevant framework for observing peptide translocation events and membrane destabilization. The method’s flexibility to simulate varying peptide-to-lipid ratios makes it more attractive for modeling cooperative and concentration-dependent effects. Together, these two techniques offer complementary insights: aSMD + cMD quantifies the energy landscape of translocation, while CompEL captures the emergent, collective dynamics that drive peptide-induced membrane remodeling.

### Summary of computational techniques

A wide range of computational techniques have been developed to probe the complex mechanisms underlying CPPs' and MAPs’ interactions with lipid bilayers. Each method offers specific techniques and is best suited for distinct research objectives. Enhanced sampling methods, such as SMD, aSMD, US, MetD, REMD, and WE, provide detailed free-energy and kinetic information but often require substantial computational resources and are not entry-level. Alternative strategies, including HT-MD, MT-MD, CG-MD, and IMM, extend accessible timescales and simplify system representation yet may sacrifice atomic resolution or physiological accuracy. Within this landscape, the methodologies presented by our laboratory, aSMD + cMD and CompEL, offer other alternatives. aSMD + cMD also leads to PMF calculation and allows for observation of peptide-membrane interactions. In parallel, CompEL enables efficient simulation of spontaneous or potential-driven translocation processes across bilayers, making it particularly suitable for exploring cooperative effects, multi-peptide systems, and membrane asymmetry.

Ultimately, the selection of an appropriate simulation technique (or combination of several) should be guided by the specific goals of the study, whether to quantify energy barriers, explore large-scale peptide-membrane dynamics, capture cooperative effects, or approximate physiological conditions. No single method is universally superior; rather, each presents a balance of resolution, computational cost, and mechanistic insight. As computational power and hybrid modeling approaches continue to advance, integrating complementary methods will deepen our understanding of peptide-driven membrane translocation and disruption. Furthermore, integrating artificial intelligence (AI) into CPP research holds great potential to revolutionize the discovery and optimization of CPPs (Tran et al. 2021; Imre et al. 2024; Fang et al. 2025; Sun et al. 2025). AI can analyze complex, multidimensional datasets to uncover hidden sequence-activity relationships and predict membrane-translocating potential with remarkable accuracy. When combined with MD simulations, AI-driven approaches can accelerate peptide design, guide experimental validation, and provide deeper mechanistic understanding of CPP-membrane interactions.

## Conclusions from computational studies

### Peptide analysis

Both aSMD + cMD and CompEL approaches provide valuable insights into the key interactions governing peptide transport across lipid membranes. Firstly, positively charged amino acids play a central role in peptide partitioning, as their electrostatic attraction to negatively charged lipid headgroups stabilizes peptide binding to the bilayer (Lopez et al. 2006; He et al. 2015; Alaybeyoglu et al. 2016; Muñoz-Gacitúa et al. 2022). Among these residues, arginine has been shown to interact more strongly with anionic headgroups than lysine, a difference attributed to the guanidinium functional group unique to arginine (Lai And Kaznessis 2018; Hao et al. 2022; Tempra et al. 2023). In addition, polar residues contribute to interactions with headgroups in the lower leaflet, which are essential for the successful completion of the translocation process. The importance of these interactions helps explain the prevalence of positively charged residues in CPPs and, more broadly, in MAPs, as they facilitate binding to polar regions on both sides of the bilayer (Avci et al. 2018; Klug et al. 2024).

At the same time, nonpolar amino acids preferentially associate with the hydrophobic core formed by lipid acyl chains, while polar and charged residues can maintain contacts with headgroups in the upper leaflet. This complementary set of interactions promotes peptide stability once inserted into the membrane. Accordingly, hydrophobic residues are also functionally important in CPPs, as they help balance peptide-membrane interactions by anchoring the peptide within the membrane interior (Lopez et al. 2006; Fuselier And Wimley 2017; Hao et al. 2022; Muñoz-Gacitúa et al. 2022).

In short, an appropriate combination of charged and hydrophobic residues, and a proper balance between them, appears advantageous for both CPPs and MAPs (Frøslev et al. 2022; Oba et al. 2023). This balance is observed in several well-characterized CPPs, including penetratin, TAT, or MAP. Oligoarginines represent a notable exception, as they can translocate efficiently despite lacking hydrophobic residues (He et al. 2015). Nevertheless, peptide composition alone does not fully determine translocation efficiency, and additional factors, such as membrane composition, peptide concentration, or peptide secondary structure, must also be taken into account.

Among these parameters, peptide concentration plays a central role in CPP translocation. Nonetheless, this variable is frequently overlooked in computational studies, largely because many simulation approaches are limited or better suited for single-peptide systems. To the best of our knowledge, only a restricted set of methodologies has been applied to systems containing multiple peptide molecules, namely cMD, HT-MD, MT-MD, and CG-MD (Herce And Garcia 2007; He et al. 2015; Wang et al. 2016).

Firstly, aSMD in combination with cMD approach restricts simulations to a single peptide molecule. Consequently, one peptide and 300 lipid molecules were used in our studies, corresponding to a peptide:lipid (P:L) ratio of 1:300. However, CompEL explicitly allows running simulations with different P:L ratios. Even though our initial CompEL simulations were conducted using one peptide per bilayer comprising 256 lipid molecules (1:256 P:L ratio), subsequent simulations incorporated up to eight peptides (1:32 P:L ratio). Previous studies have demonstrated that increasing the number of peptides does not significantly alter the free energy barrier for insertion (MacCallum et al. 2011). At the same time, higher peptide numbers are known to induce stronger membrane perturbations, which are often required to facilitate peptide insertion and translocation (Leontiadou et al. 2006; Herce And Garcia 2007; Irudayam And Berkowitz 2012; He et al. 2015; Bartoš et al. 2021; Choe 2023; Rice et al. 2025).

Accordingly, additional CompEL simulations were performed at a higher P:L ratio of 1:32. Under these conditions, a greater number of insertion and translocation events were observed, even though typically only one or two peptides successfully inserted into the membrane (Leontiadou et al. 2006). This behavior highlights the importance of peptide cooperativity, since multiple peptides collectively enhance bilayer perturbation. Moreover, insertion of one peptide can lower the energetic barrier for subsequent peptide entry (Illya And Deserno 2008). Importantly, elevated P:L ratios more closely resemble experimental conditions, in which relatively high peptide concentrations are commonly employed (Walrant et al. 2013; Manzini et al. 2014). Such conditions are also physiologically relevant, as peptides can accumulate at membrane surfaces due to electrostatic attraction, leading to local concentrations that far exceed bulk values (Svirina And Terterov 2021). Thus, simulations performed at higher peptide concentrations not only improve comparability with experimental data but also provide a more realistic representation of peptide-membrane interactions in biological contexts.

Experimental evidence further supports the critical role of peptide concentration in CPP translocation. For example, Binder and Lindblom (2003) demonstrated that penetratin internalization requires a P:L ratio exceeding approximately 1:20. Below this threshold, penetratin remains bound to the vesicle surface, whereas higher peptide concentrations enable transbilayer transport. Their findings suggest that the accumulation of positively charged peptides at the membrane surface generates a transmembrane electric field, promoting membrane permeabilization and peptide translocation. Following peptide internalization, the dissipation of this electric field restores membrane integrity, where an applied Δ*Ψ* accelerates membrane permeabilization. Similar concentration-dependent effects have been reported for other CPPs. The TAT peptide, for instance, requires relatively high concentrations to induce membrane disruption and promote the formation of transbilayer water channels (Chen et al. 2013). Nevertheless, penetratin has also been shown to internalize at low concentrations in certain experimental conditions (Su et al. 2008), indicating that translocation mechanisms and cooperative effects vary substantially among CPPs and depend strongly on peptide sequence and local peptide density.

A second important variable is the peptide orientation throughout the simulation. The peptide orientation varies as it crosses the bilayer (Yue et al. 2016; Kabelka and Vácha 2018). During the initial partitioning phase, polar residues interact primarily with lipid headgroups or surrounding water molecules, while hydrophobic residues are shielded from polar environments through intrapeptide interactions. In this state, peptides usually remain largely parallel to the bilayer. Upon insertion into the membrane, hydrophobic residues engage with the membrane core, while interactions between positively charged residues and lipid headgroups in the upper leaflet are largely preserved. Simultaneously, the peptide starts interacting with the lipid polar heads present in the lower leaflet. As a result, polar residues interact with both leaflets, while hydrophobic residues remain centered within the membrane, causing the peptide to elongate and orient perpendicularly to the bilayer. Successful translocation requires disruption of interactions with the upper leaflet and the membrane core, forcing hydrophobic residues to shield themselves again from polar environments and leading the peptide to reorient parallel to the bilayer plane.

A third factor contributing to membrane disruption is peptide secondary structure. Previous studies have shown that peptides capable of adopting α-helical conformation often display enhanced transcellular transport efficiency (Hao et al. 2022; Oba et al. 2023). However, peptide secondary structure is highly dynamic and depends on both amino acid composition and interactions with the membrane environment (Yue et al. 2016).

In our CompEL study (Catalina-Hernandez et al. 2025a), Arg9, which lacks hydrophobic residues, remains largely unstructured, favoring interactions with polar components and predominantly adopting coil or turn conformations. Leu9, by contrast, preferentially interacts with hydrophobic regions and adopts an α-helical conformation upon insertion into the pore, with hydrophobic sidechains facing outward and polar backbone atoms oriented inward to interact with water molecules. TP10 is unique in that it already exhibits α-helical structure in our simulations in water and maintains this conformation during CompEL simulations. Notably, in systems containing eight peptides, TP10 gains additional helical content, resembling the behavior observed for Leu9 upon membrane interaction. Similarly, TP2 adopts α-helical structure in CompEL simulations with eight peptides, consistent with previous reports describing secondary structure changes upon membrane insertion (Bennett et al. 2024).

Finally, it is important to note that membrane composition can significantly influence peptide secondary structure (Meher And Chakraborty 2019). Therefore, future computational studies should incorporate membrane heterogeneity as an additional variable when investigating CPP-membrane interactions.

### Membrane analysis

Membrane lipid composition plays a decisive role in modulating peptide insertion and translocation. Across the simple bilayer models examined (Catalina-Hernandez et al. 2025a, b, c), namely DPPC, DOPC, and POPC, no substantial differences were observed, as all three membranes supported peptide insertion and translocation events. In contrast, incorporation of cholesterol reduced the frequency of both insertion and full translocation (Catalina-Hernandez et al. 2025b, c), in agreement with earlier computational and experimental studies (Sapay et al. 2009; Pae et al. 2014; Maia et al. 2025). A similar inhibitory effect was observed upon the inclusion of negatively charged lipids in both the upper and lower leaflets, with a particularly pronounced reduction in complete translocation events. This behavior suggests that peptides preferentially interact with anionic lipid headgroups in the upper leaflet, which stabilizes surface binding and diminishes further membrane disruption (He et al. 2015; Jain And Matysiak 2024). Although the presence of cholesterol and variations in lipid tail composition did not lead to marked increases in the PMF, the introduction of negatively charged lipid species in the upper leaflet resulted in a noticeable increase in the energetic barrier for bilayer crossing. Collectively, these findings reinforce the notion that CPP translocation is highly sensitive to membrane lipid composition (Richard et al. 2005).

Beyond translocation efficiency, peptide-membrane interactions can also be considered in terms of their impact on bilayer structural properties, including membrane thickness and lipid order parameters (*S*_CH_, which analyses the orientation of lipids regarding the membrane normal, or whether lipids are correctly ordered in the membrane) (Catalina-Hernandez et al. 2025b, c). Simple membranes (DPPC and DOPC) exhibit comparable thickness values of approximately 37–38 Å. In contrast, membranes containing both cholesterol and mixed lipid tails, DPPC:DOPC:CHOL, displayed increased thickness values of around 42 Å. This observation suggests that differences in acyl chain composition hinder optimal lipid packing, leading to bilayer thickening. When negatively charged lipids were added to these systems (DPPC:DOPC:DOPS:CHOL and DPPC:DOPC:DPPS:DOPS:CHOL), enhanced intermolecular interactions promoted tighter packing (He et al. 2015; Jain And Matysiak 2024) and resulted in a modest reduction in membrane thickness to approximately 40 Å. Importantly, comparison with peptide-free control simulations revealed that peptide presence did not significantly alter bilayer thickness, indicating that these structural changes arise primarily from lipid composition rather than peptide insertion.

Lipid order parameter analyses show that membranes remained globally well ordered, even in the presence of peptide insertion, pore formation, or translocation. This apparent preservation of overall membrane order can be explained by spatial compensation: while lipid order decreases locally in the vicinity of the peptide or pore, it increases in regions distant from these perturbations, thereby maintaining the average bilayer order (Leontiadou et al. 2006).

Following membrane disruption events, particularly those involving transient pore formation, the bilayer generally tended to reseal and return to a state resembling its equilibrated configuration. In some cases, however, lipids initially dragged from the upper leaflet during peptide interaction became stabilized in the lower leaflet, resulting in lipid flip-flop events (Catalina-Hernandez et al. 2025a). Previous studies have identified pore formation as a prerequisite for lipid flip-flop (Lai And Kaznessis 2018), and other reports have demonstrated that such events often occur concurrently with peptide insertion or translocation (Tieleman and Marrink 2006; Vernier et al. 2006; Muñoz-Gacitúa et al. 2022; Choe 2023). Moreover, lipid flip-flops are generally observed from the upper to lower leaflet, supporting earlier findings that flip-flops preferentially occur from peptide-enriched leaflets toward peptide-free regions of the membrane (Leontiadou et al. 2006).

## Applications

CPPs have emerged as versatile tools in biomedical research and therapeutic development owing to their exceptional capacity to transport diverse molecular cargoes across cellular membranes. One of their principal applications lies in drug delivery, where CPPs enable the intracellular transport of small molecules, peptides, proteins, nucleic acids, and drugs. By facilitating cellular entry, CPPs enhance bioavailability and therapeutic efficacy, particularly for compounds that otherwise exhibit poor membrane permeability (Datta 2025).

In the context of gene therapy, CPPs serve as non-viral vectors for the delivery of genetic materials aimed at treating inherited or acquired disorders. Compared to viral systems, they offer advantages such as reduced immunogenicity and improved safety profiles, making them attractive alternatives for clinical use (Hoyer And Neundorf 2012; Gagat et al. 2017; Geng et al. 2022). CPPs have also shown promise in oncology, where they enable the targeted delivery of chemotherapeutics and biologics, mitigate systemic toxicity, overcome multidrug resistance, and act as tumor-specific imaging probes (Gori et al. 2023).

Additionally, CPPs are being explored in vaccine design to enhance antigen uptake, intracellular processing, and presentation, thereby promoting stronger and more durable immune responses (Brooks et al. 2010). Other notable applications include treatments for inflammatory diseases through transdermal delivery, antimicrobial therapy, and incorporation into advanced nanocarrier platforms, such as liposomes, nanoparticles, dendrimers, and exosomes, to improve targeted delivery and therapeutic performance (Datta 2025).

Beyond their use as delivery vectors, several CPPs function as therapeutic or diagnostic agents. For example, the CPP p28 binds to DNA-binding domains, inhibiting the proliferation of cancer cells, while fluorescently labeled CPPs can assist in intraoperative tumor visualization, thereby increasing the precision of surgical resections (Nguyen et al. 2010; Yamada et al. 2013).

## Challenges and take-home messages

Although CPPs hold considerable promise as molecular delivery vectors, several challenges continue to limit their clinical and experimental utility, such as limited target specificity (most CPPs enter a broad range of cell types, risking off-target effects that are particularly concerning in oncology) together with dose-dependent cytotoxicity that can mirror AMP-like membrane disruption at higher concentrations (Jauset And Beaulieu 2019; Datta 2025; Umezawa et al. 2025). Proteolytic instability reduces plasma half-life, and while cyclization, PEGylation, or non-natural residues improve stability, they may diminish permeability or introduce immunogenicity (Kristensen et al. 2016). Endosomal sequestration frequently traps CPP-cargo complexes, and chemical strategies aimed at promoting endosomal escape can inadvertently raise toxicity (Madani et al. 2011; Reissmann 2014; Sahni et al. 2020). Practical barriers (scale-up and cost of synthesis, extensive toxicology requirements, and analytical detection at low concentrations) further complicate translation (Kristensen et al. 2016; Khakshur et al. 2025). Despite decades of research demonstrating remarkable membrane interaction and translocation capacities, clinical impact remains modest, underscoring the need to more sharply connect physicochemical mechanisms with design rules for selectivity, efficacy, and safety.

From a computational perspective, three take-home principles can help reconcile mechanism with method choice and make results more comparable across studies. First, adopt a multi-scale workflow: leverage techniques that allow cooperative behavior (e.g., CompEL), explore different membrane compositions, correlate simulations with experimental data when possible, and compute PMFs using US technique. However, when the primary objective is to observe peptide-induced pore formation and nucleation, CG-MG should be employed to access the longer timescales required for these events. The CompEL + US sequence balances realism, mechanism discovery, and quantitative comparability (Kutzner et al. 2011; Ouyang et al. 2022). Two cautions follow: (i) absolute barriers are method-dependent, as non-equilibrium aSMD tends to report higher PMFs than US unless bracketed with equilibrium windows; and (ii) the reaction-coordinate definition (full-bilayer translocation vs. insertion to the midplane) can shift reported energies and even alter peptide rankings (Torrie And Valleau 1977; Catalina-Hernandez et al. 2025c). Mechanistically, the fate of the pore is a practical discriminator: CPPs typically engage the lower leaflet and allow pores to close, whereas AMPs more often stabilize pores and sustain leakage, an in silico phenotype that aligns with biological function (Alimohamadi et al. 2023; Rice et al. 2025).

Second, enable meta-analysis through benchmarking and reporting standards. Authors should standardize and disclose membrane recipes (cholesterol/anionic lipid fractions; leaflet asymmetry), P:L ratios, applied Δ*Ψ* (when relevant), CV definitions, pulling speeds, and the PMF reference state (e.g., water vs. bilayer center). As a default, bracket aSMD-derived PMFs with a small set of US windows at key depths and explicitly state whether the PMF spans full translocation or insertion only (Torrie And Valleau 1977; Catalina-Hernandez et al. 2025c). These steps prevent common cross-study discrepancies arising from force protocols and coordinate choices (Kutzner et al. 2011; Ouyang et al. 2022).

Third, apply actionable decision rules: (1) Electrostatics-dominated (Arg-rich) systems usually follow pore-mediated routes; use CompEL/aSMD + cMD for mechanism and US for PMFs. (2) Hydrophobic-dominated systems tend toward insertion; atomistic MD may suffice when timescales are accessible, while CG simulations (followed by CG → AA back-mapping) are appropriate when insertion involves slow, collective lipid rearrangements. (3) Δ*Ψ*, cholesterol, and anionic lipids lower pore-formation thresholds and can shift the dominant route. (4) When mechanistic ambiguity remains, combine aSMD + cMD (path sampling) with US (quantitativebarrier). (5) AI is valuable for triage but must address negative-set scarcity; its role is to prioritize sequences for mechanistic MD and PMF quantification rather than to replace them (Rothbard et al. 2004; Ouyang et al. 2022; Ramasundaram et al. 2024).

Looking ahead, community-level actions can accelerate convergence and translation: standardize membrane compositions (including asymmetry), P:L ratios, and Δ*Ψ* usage; publish CVs, pulling speeds, and PMF reference states; establish benchmark sets that link AI predictions to MD-observed mechanisms; and treat complex membranes (asymmetry, cholesterol, anionic lipids) as routine rather than exceptional (Kutzner et al. 2011; Ouyang et al. 2022). Implementing these low-cost practices will make computational findings more reproducible and mechanistically coherent, ultimately informing the rational design of next-generation CPPs with improved target selectivity, safety profiles, and delivery performance. At the same time, deeper insight into CPP internalization will continue to illuminate fundamental aspects of membrane biology and support progress in targeted therapeutics, molecular diagnostics, and precision medicine (Moreno-Vargas And Prada-Gracia 2024).

## Data Availability

No datasets were generated or analysed during the current study.
